# A Review of the Mechanical Behavior of Magnesium Alloys in Compression: From Mechanistic Competition to Structural Regulation

**DOI:** 10.3390/ma19101966

**Published:** 2026-05-10

**Authors:** Qinghui Zhang, Shuchen Wang, Yiming Ma, Xuehua Li, Zhijun Li, Xianzhe Shi

**Affiliations:** 1 School of Civil & Architecture Engineering, Xi’an Technological University, Xi’an 710021, China; zhangqinghui_6@163.com (Q.Z.); 15332234526@163.com (S.W.); 15339053045@163.com (Y.M.); 2 Aerospace College, Northwestern Polytechnical University, Xi’an 710021, China; shixianzhe@nwpu.edu.cn

**Keywords:** magnesium alloys, compressive deformation, slip, twinning, dynamic recrystallization, gradient structures

## Abstract

Magnesium alloys that are low density and have a high specific strength are widely utilized as lightweight structural materials. Due to their hexagonal close-packed crystal structure, plastic deformation in magnesium alloys is strongly limited in dislocation slip and mainly accommodated by deformation twinning, which results in distinct mechanical anisotropy and tension–compression asymmetry. This paper, centered on mechanism competition and microstructure regulation, systematically reviews the recent progress in the compressive mechanical responses of magnesium alloys. Key results reveal the cooperative and competitive mechanisms between slip and twinning, the significant controlling effects of temperature and strain rate on deformation behavior, and the effective design strategies of gradient and heterogeneous structures that achieve superior strength–ductility synergy. This review provides essential theoretical support for the development and performance optimization of high-performance magnesium alloys.

## 1. Introduction

Magnesium alloys are among the most promising lightweight structural metals, valued for their ultra-low density and exceptional specific strength and stiffness [1,2,3,4]. They have been widely used in biomedical implants, electronics, and in the automotive and aerospace industries. With the increasing focus on energy conservation, emission reduction, and lightweight manufacturing, green processing of magnesium alloys has become a key research trend. At the same time, emphasis on their mechanical properties remains critical as many application fields impose strict requirements on their performance. Notably, their low density and high specific strength make magnesium alloys ideal for civil and military ballistic impact protection structures, high-speed stamping, and automotive crash applications. These fields specifically demand that magnesium alloys can withstand high-strain-rate compressive conditions and exhibit excellent mechanical properties, underscoring the significance of prioritizing mechanical performance in the research and application of magnesium alloys.

Magnesium grains exhibit a hexagonal close-packed (HCP) crystal structure. At room temperature, plastic deformation is dominated by basal slip, while non-basal slip systems (prismatic and pyramidal) are difficult to activate under low stress owing to their high critical resolved shear stress (CRSS) and large activation threshold [5]. Consequently, magnesium alloys suffer from poor ductility, which limits their wider application. Moreover, the asymmetric HCP structure easily leads to the formation of strong crystallographic texture during processing, and the preferred orientation of polycrystals significantly affects mechanical properties and deformation mechanisms [6]. When loading direction deviates from the lattice c-axis, different slip and twin systems are activated. The large difference in CRSS for various deformation systems results in distinct mechanical anisotropy of magnesium alloys [7,8]. Meanwhile, the different lattice strain states and activated deformation mechanisms (especially twinning) under tension and compression lead to significant tension–compression asymmetry. Both anisotropy and asymmetry severely restrict the ductility and formability of magnesium alloys.

In practical engineering, magnesium alloy components usually serve in complex external environments, such as high-strain-rate loading and multiaxial stress states, which directly affect the load-bearing capacity and service life. Investigating mechanical properties under simplified loading conditions provides a fundamental basis for evaluating material behavior under complex loading and guiding engineering applications. Magnesium alloys are commonly used in high-speed stamping, ballistic protection structures, and accidental automotive loading, where understanding their compressive mechanical behavior is particularly critical. Under compression, c-axis strain cannot be effectively accommodated by basal slip, so tensile twins are preferentially activated. The large lattice rotation caused by twinning reconstructs crystallographic orientation and further influences texture evolution and mechanical properties [9,10,11]. However, excessive twinning promotes work hardening and local stress concentration that reduce ductility, while low CRSS of tensile twins reduces yield strength. Therefore, improving the compressive mechanical properties of magnesium alloys is essential to resist high-speed impact damage in engineering.

Great efforts have been made to strengthen and toughen magnesium alloys through alloying, thermomechanical processing, and microstructure modification. This paper reviews the research progress on compressive mechanical behavior of magnesium alloys following the logical framework of “mechanism–response–design”. The fundamental deformation mechanisms and the coupling relationship between slip, twinning, and texture evolution are introduced first. Then, the compressive responses regulated by temperature, strain rate, and loading direction are analyzed. Furthermore, performance optimization strategies, including gradient/heterogeneous structures, second-phase strengthening, twinning regulation, and grain refinement, are summarized. Finally, the advances in multiscale modeling and data-driven design of magnesium alloys are reviewed and prospected. This systematic review aims to provide theoretical support and design principles for the performance enhancement and engineering applications of magnesium alloys.

## 2. Fundamental Deformation Mechanisms of Magnesium Alloys

**Figure 1:**
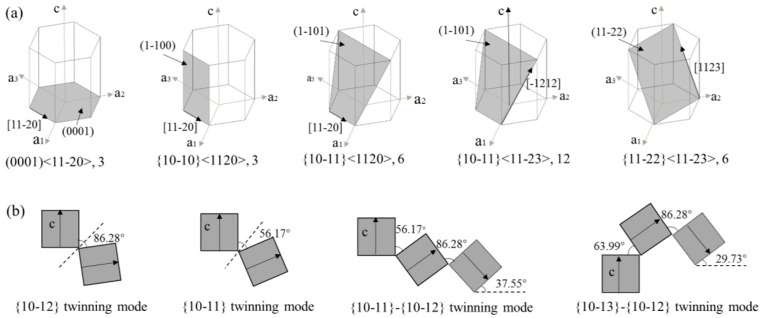
(**a**) twinning systems in magnesium alloys; (**b**) Slip systems in magnesium alloys [12].

The HCP crystal structure of magnesium grains is characterized by the axis ratio c/a = 1.624, with lattice parameters a = 0.3209 nm and c = 0.5211 nm. As shown in Figure 1a, magnesium has several distinct slip systems, including the (0001) basal slip system, the {10-10} prismatic slip system, and the {10-11} pyramidal slip system. Only the {0001}<11-20> basal slip system is readily activated and predominant during ambient-temperature plastic deformation, since its (0001) plane is the most densely packed atomic plane, <11-20> is the most densely packed atomic direction [12], and it has the lowest critical resolved shear stress (CRSS) [1,2]. Basal slip only provides three independent slip systems, making it challenging to meet the von Mises criterion (requiring at least five independent slip systems for uniform plastic deformation) [13]. Additionally, basal and prismatic slips only accommodate an in-plane deformation of (0001) and cannot coordinate deformation along the lattice c-axis, thus requiring pyramidal slip or twinning [14]. Prismatic slip ({10-10}<11-20>), with higher CRSS than basal slip, can be activated under suitable temperature and stress, synergizing with twinning to enhance plasticity [15,16]. Li et al. [15] reported prismatic slip as the main deformation feature in weakly textured Mg–1Al–0.5Mn–0.3Gd alloys. Specifically, under tension, it causes higher rheological stress along the rolling direction (RD) than the transverse direction (TD), and under compression, it cooperates with twinning to adjust grain orientation, reduce strain concentration, and improve plastic coordination [16]. In contrast, Feng et al. [11] found pyramidal slip ({10-11}<11-20>) dominates the high-strain stage of uniaxial compression in rare earth Mg–Y–Al–Ca–Mn alloys, significantly enhancing plasticity. However, these studies [11,15] reveal an inconsistency: prismatic slip is dominant in Gd-containing alloys, while pyramidal slip prevails in Y-containing alloys. This discrepancy likely stems from rare earth type and content that alters non-basal slip. Specifically, CRSS–Gd may preferentially reduce prismatic slip, while Y has a greater effect on pyramidal slip. A critical knowledge gap is the lack of systematic studies comparing the effects of rare earths on non-basal slip activation, which hinder high-plasticity magnesium alloy design.

Non-basal slip is more easily triggered by higher loading stress or temperature [17]. To achieve uniform plastic deformation, researchers focus on promoting multi-slip system activation via alloy composition adjustment. Patel et al. [18] found Gd and Y alloying modifies CRSS to regulate slip and twinning systems. Mirzakhani and Assempour [19] confirmed that Y enhances Mg–Y alloy strength and ductility by weakening texture, reducing basal slip dominance, and promoting non-basal slip and twinning.

As seen in Figure 1b, common deformation twins in magnesium alloys include {10-12} tensile twins, {10-11}/{10-13} compressive twins, and {10-11}-{10-12}/{10-13}-{10-12} double twins [20]. Tensile twinning occurs when the grain’s lattice c-axis is subjected to tensile stress (e.g., compression along the basal plane or normal stretching perpendicular to the basal plane), while compressive twinning is induced by compression perpendicular to the basal plane, involving a 57° lattice rotation around the <11-20> axis [21]. Secondary tensile twins often form within regions of compressive twins [22]. The sequential occurrence of these two twinning mechanisms generates double twins, with the lattice in the secondary twin region rotating an additional 38° around the same axis. Figure 1 is critical for understanding the deformation characteristics of magnesium alloys. Specifically, Figure 1a illustrates the three primary slip systems, with the basal slip system’s low critical shear stress (CRSS) explaining its dominance at ambient temperature, while the prismatic and pyramidal systems’ higher CRSS limits their activation under mild conditions. Figure 1b depicts the main twinning modes, highlighting that {10-12} tensile twins and {10-11} compressive twins are the most functionally relevant for c-axis strain coordination. Together, these systems determine the alloy’s plastic deformation capacity and anisotropic behavior.

Twinning is a low-energy, fast-responding deformation mode when basal slip is inhibited. Tensile twinning is the most common mode under compression [23], enabling ~86.3° orientation rearrangement to accommodate c-axis strains [15,16] and reorienting grains to favor basal slip when the c-axis is parallel to compression [24], though excessive twinning increases work hardening at the cost of ductility. {10-11} compressive twins form preferentially under c-axis compression or high stress, which accounts for their high proportion in coarse-grained alloys or in large plastic deformation. Moreover, their volume fraction increases with strain, affecting texture evolution and strain hardening [5]. In situ EBSD and mechanical tests [5] show that uniaxial compressive strain promotes {10-11} twin growth and multiplication, inducing secondary twinning and complicating deformation paths. Under complex fatigue/cyclic loading, laminar secondary twins (e.g., {10-11}-{10-12}, {10-12}-{10-12}) are common, influencing deformation localization, crack initiation, and damage evolution.

Twinning increases slip channels and exhibits a “competition synergy” with slip to coordinate plastic deformation: twin boundaries inhibit dislocation motion to enhance work hardening and strength, while in slip-limited orientations, twins first rearrange crystal orientations to enable more slip system participation [2,10]. Regulating twin volume fraction and distribution improves ductility. This is shown by Chen et al. [4], who controlled basal texture divergence and tilt to inhibit excessive twinning, promote slip-dominated deformation, and reduce tensile–compressive asymmetry. Yang et al. [10] noted that adjusting the twinning ratio directly affects an AZ31 magnesium alloy’s hardening behavior and plastic response.

Different slip and twin systems within magnesium alloy grains participate in plastic deformation under external loading, and their activation and interaction significantly influence the material’s mechanical behavior and texture evolution [1,2,3,4,5,9,10]. However, the CRSS required to activate different twin and slip systems varies drastically. The CRSS for basal slip is the smallest, while the CRSS for compressive twinning is the largest [25,26]. This significant disparity in CRSS values leads to competing activation of deformation mechanisms under different loading conditions, a topic where minor inconsistencies exist in the literature. For example, some studies [12,13] emphasize basal slip as the sole dominant mechanism at ambient temperature, while others [16] suggest prismatic slip can be partially activated under mild compression, especially in weakly textured alloys. These inconsistencies may stem from differences in alloy composition (e.g., rare earth additions) and initial texture, which alter CRSS values and slip system activation thresholds. The extremely low CRSS of basal <a> slip (0.45–0.8 MPa) explains its dominance at ambient temperature, while the much higher CRSS of prismatic (39.2 MPa) and pyramidal (45–81 MPa) slip systems means they only activate under higher stress or temperature. Notably, {10-11} compressive twinning has the highest CRSS (76–153 MPa), indicating that it only forms under severe c-axis compression or high strain rates. This explains why tensile twinning ({10-12}, CRSS = 2.0–2.8 MPa) is more common under moderate compression. These values also highlight a critical knowledge gap: the effect of alloying elements (e.g., Gd, Y) on CRSS variations is not fully quantified, with conflicting reports on how rare earth additions alter the relative activation of slip vs. twinning [18,19].

Under varying loading situations, magnesium alloys exhibit distinct plastic deformation mechanisms. In addition, texture is easily formed during magnesium alloy processing and molding, and this texture has a substantial impact on the material’s mechanical properties and deformation mechanisms. The formation of texture is primarily governed by the synergistic evolution of slip and twinning; the competition and alternation between these two mechanisms drive the gradual orientation concentration, eventually leading to the development of a strong basal texture [27,28,29,30]. Texture serves as a crucial link between deformation mechanisms and macroscopic properties: tensile twinning shifts the c-axis to be almost orthogonal to the compressive direction, while basal slip deflects the c-axis in the tensile direction. Dynamic recrystallization (DRX) can weaken plastic anisotropy by breaking the initial texture concentration and generating a more uniform orientation distribution [29,30]. Magnesium alloys are typically processed and molded at high temperatures because their deformation coordination ability is poor at room or low temperatures. Twinning-induced recrystallization (TIR) is a key mechanism affecting texture evolution, but inconsistencies exist in the literature regarding its effect. Some studies [27,28] report that TIR reduces the strength of the original texture and forms new texture clusters, while others suggest TIR has little effect on texture strength in rare earth-containing alloys. This discrepancy may be related to alloy composition and deformation temperature such that rare earth elements may inhibit grain rotation during TIR, preserving the original texture.

Furthermore, under specific temperature and strain-rate conditions, magnesium alloys undergo DRX, which modifies the material’s macroscopic mechanical properties by refining grains and weakening texture. As a crucial softening mechanism during thermal processing, DRX in magnesium alloys mainly includes discontinuous dynamic recrystallization, continuous dynamic recrystallization, twinning-induced recrystallization, and particle-stimulated nucleation [11,27,31,32,33,34,35,36]. Discontinuous recrystallization typically nucleates at grain boundaries and gradually replaces parent grains via boundary migration [33,34]. Continuous recrystallization develops through the formation and rotation of subgrain boundaries, eventually transforming into high-angle boundaries [34]. Twinning-induced recrystallization frequently initiates near twin boundaries, revealing the coupling between twinning and recrystallization [27]. Particle-stimulated nucleation is driven by stress concentration around second-phase particles [31,35], while rotational recrystallization may occur under severe plastic deformation [36]. These processes are highly sensitive to temperature, strain rate, composition, and initial grain size [37,38,39,40]. Higher temperatures, moderate strain rates, and fine-grained microstructures favor sufficient DRX and homogeneous grain structures. Rare-earth alloying and second-phase particles further regulate DRX behavior. For instance, the Mg_3_Bi_2_ phase pins grain boundaries and inhibits grain growth [39], whereas the I-phase transformation in Mg–Zn–Y alloys weakens the confinement of recrystallization [38].

In summary, at room temperature, deformation of magnesium alloys is dominated by slip and twinning, which govern texture evolution and determine macroscopic mechanical response, laying an important foundation for property-oriented material design. At elevated temperatures and controlled strain rates, DRX operates synergistically with slip and twinning to accommodate deformation and further tailor the mechanical performance of magnesium alloys.

## 3. Compressive Mechanical Behavior of Magnesium Alloys

Building on the deformation mechanisms discussed in Section 2, this section focuses on the compressive mechanical behavior of magnesium alloys, which is closely related to the activation and interaction of slip, twinning, and DRX. Magnesium alloy grains have an asymmetric HCP crystal structure and exhibit high sensitivity to external conditions during compressive deformation. The most critical control variables are temperature, strain rate, and loading direction, which collectively influence the activation sequence and relative contributions of slip and twinning [41], and thereby alter the rheological stress, work-hardening rate, and microstructural stability [42,43,44,45,46,47,48,49,50].

### 3.1. Influence of Loading Direction

The influence of loading direction on the compressive mechanical properties of magnesium alloys is mainly attributed to the material’s initial texture [51]. The orientation of polycrystals in the raw material exhibits a certain distribution, and when the loading direction deviates from the c-axis of most grains, various slip and twin systems are activated during plastic deformation. The significant difference in CRSS required to activate different slip and twin systems leads to pronounced differences in mechanical properties under different loading directions, highlighting the alloy’s anisotropic characteristics [7,8,52]. Deformation along the c-axis is primarily coordinated by twinning: under compression, {10-11} compressive twins (high CRSS) are activated, resulting in high flow stress and relatively low strain hardening. In contrast, under tension, {10-12} tensile twins (low CRSS) dominate the initial deformation stage, leading to low-flow stress. As dislocation–dislocation and dislocation–twin interactions intensify, the strain hardening rate increases significantly in the later deformation stage.

**Figure 2:**
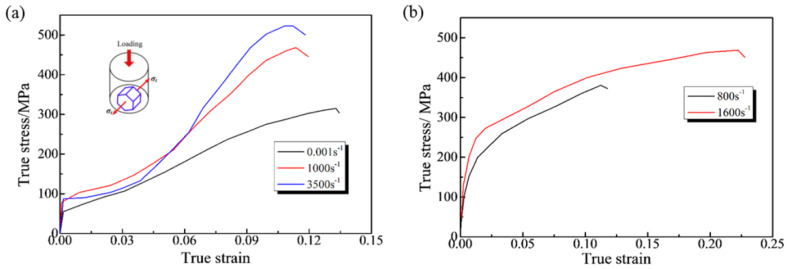
True stress−strain curves of an AZ31 magnesium alloy under compressive loading at different strain rates. (**a**) The compression direction is perpendicular to the crystal grain’s lattice c-axis [25]. (**b**) The compression direction is parallel to the crystal lattice c-axis of the crystal grain [26].

Figure 2 illustrates the compressive stress–strain curves of the AZ31 magnesium alloy under normal direction (ND) loading at different strain rates. The loading conditions in Figure 2a,b are identical, but the materials have different texture structures, resulting in different mechanical properties. Figure 2a shows the compressive mechanical properties of an AZ31B magnesium alloy extruded plate under high-strain-rate loading in the ND direction. After extrusion, the c-axis of most grains is inclined to the transverse direction so that the compressive loading direction is orthogonal to the c-axis. This induces tensile deformation and activates {10-12} tensile twins initially, explaining why yield stress is insensitive to strain rate. As strain increases, pyramidal slip dominates, making strain hardening rate-sensitive and leading to an “S”-shaped stress–strain curve. In contrast, Figure 2b shows the compressive properties of AZ31B with most grain c-axes aligned with the ND direction. C-axis compression during impact activates basal/pyramidal slip and {10-11}-{10-12} twins (high CRSS), resulting in strain rate-sensitive flow stress. These curves clearly demonstrate how texture (grain c-axis orientation) governs the activation of deformation mechanisms, and thus, compressive behavior.

Dixit et al. [53] investigated the microstructural evolution and deformation mechanisms of pure magnesium under high-strain-rate compression (10^3^ s^−1^) using EBSD and TEM. The findings indicated that when the compressive axis is perpendicular to the c-axis of the lattice, numerous tensile twins form in the initial deformation stage, and twinned regions facilitate slip to accommodate plastic deformation. Additionally, dislocation density increases significantly with strain, intensifying dislocation–dislocation interactions and increasing the strain hardening rate. Yang et al. [54] examined the deformation mechanisms of magnesium alloys under dynamic compressive loading from different orientations, finding that twinning is the predominant deformation mechanism when compressed along the RD direction, while basal and non-basal slip are the primary mechanisms when compressed along the ND direction.

### 3.2. Effect of Temperature

Temperature significantly influences the mechanical properties and deformation mechanisms of magnesium alloys. As temperature increases, the CRSS of non-basal slip systems decreases, altering the relative activation of slip, twinning, and DRX. This is a key factor governing compressive behavior. Wang et al. [42] investigated the compressive deformation mechanisms of an AZ91 alloy at different temperatures, finding that deformation at low temperatures involves slip, twinning, and shear banding, whereas at high temperatures (350 °C), recrystallization, nucleation, and growth are promoted. Increasing the compression temperature significantly reduces flow stress and improves deformation stability. Zhou et al. [43] studied the warm forming and curling behavior of an AZ31 alloy and reported that twinning activity is significantly weakened at elevated temperatures, resulting in reduced bending anisotropy of the sheet. Zheng et al. [44] noted that at high temperatures, the strain–coordinating role of twins is replaced by non-basal slip, reducing the contribution of twinning. Zhang et al. [45] used molecular dynamics simulations to study the effect of temperature on the compressive deformation of nanocrystalline magnesium. They found that dislocation bundles tend to form at low temperatures while grain boundary resistance and grain reorientation dominate at high temperatures, making twinning stress-insensitive. This suggests that the effect of temperature on twinning behavior may be related to grain size and loading conditions. Therefore, magnesium alloys deform mainly via basal slip accompanied by twinning at low temperatures, whereas prismatic and pyramidal slip become increasingly active at high temperature, leading to a remarkable improvement in overall plasticity.

Within the hot deformation range (typically 300–400 °C), DRX acts as the primary softening mechanism. Increasing temperature accelerates dislocation accumulation and grain boundary migration. When stored energy reaches a critical level, new grains begin to nucleate and grow. Guo et al. [48] reported that obvious recrystallized microstructures appear in an AZ31 magnesium alloy above 350 °C, with grain size inversely correlated with the Zener–Hollomon parameter. Shen et al. [49] observed that flow stress decreases significantly in an AZ31B alloy deformed above 320 °C, with the stress–strain curve showing typical DRX characteristics. Elevated temperatures also promote twin elimination and texture randomization, leading the stress–strain curve to approach a steady state.

In summary, temperature affects deformation behavior through three main pathways: reducing the critical resolved shear stress of each slip system and promoting coordinated activation of multiple slip systems; suppressing twinning and reducing deformation anisotropy; and promoting dynamic recrystallization to refine grains, weaken texture, and enhance plasticity. Collectively, these microstructural mechanisms result in reduced flow stress and significantly improve failure strain and compressive plasticity at elevated temperatures.

### 3.3. Effect of Strain Rate

Strain rate regulates dislocation buildup and stress release, thereby affecting the contributions of slip, twinning, and dynamic recrystallization (DRX) to deformation. Higher strain rates lead to more severe dislocation plugging, enhanced work hardening, elevated rheological stress [55,56,57], increased twin formation, and greater non-basal slip involvement, with plastic coordination shifting toward slip dominance [58]. Yang et al. [56] showed that non-basal slip and deformation twinning jointly govern the deformation of Mg–Y alloys under impact, enhancing yield strength and hardening rate. Jin et al. [57] reported that a WE43 alloy at 400 °C and 10^2^ s^−1^ exhibits slip–DRX synergy, improving strength and ductility. Higher strain rates accelerate DRX nucleation via rapid dislocation energy accumulation but suppress grain growth, forming finer DRX grains with better strength and uniformity [31,32,49].

Strain rate sensitivity (SRS) determines the dynamic mechanical response under impact, correlating with crystal structure [59]. For BCC metals, SRS reflects the yield stress–strain rate relationship; for FCC metals, it relates to the strain hardening rate, with yield stress unchanged [60]. HCP metals (e.g., magnesium alloys) have complex SRS, associated with both yield stress and strain hardening. CRSS of tensile twinning and basal slip is strain rate insensitive, so dynamic properties are unaffected when these dominate deformation. In contrast, prismatic and pyramidal slip CRSS are strain rate sensitive (prismatic slip more so), leading to pronounced SRS in dynamic properties when these slip modes dominate.

## 4. Strengthening Mechanisms for Magnesium Alloy Compressive Properties

The engineering fields frequently necessitate materials capable of withstanding high-strain-rate compressive conditions and those that exhibit superior mechanical characteristics. The improvement of magnesium alloy performance now relies on a combination of strengthening methods, moving towards the synergistic optimization of strength and plasticity via multiscale microstructural manipulation. Currently, grain refinement, second-phase and twin strengthening, and gradient/heterostructure design are the main methods for improving compressive properties [36,61,62,63].

### 4.1. Grain Refining Strengthening

The Hall–Petch equation [64] delineates the relationship between yield strength and grain size in metallic materials: $\sigma_{y}=\sigma_{0}+kd^{-1/2}$, where $\sigma_{y}$ represents the yield stress of polycrystals; $\sigma_{0}$ denotes the frictional or internal stress, indicative of the grain’s resistance to deformation and can be interpreted as the material’s yield stress; and *k* signifies the grain boundary resistance, illustrating the influence of grain boundaries on deformation, which is associated with the crystal structure. The magnitude of *k* is proportional to the square of the Taylor factor *M*, and *d* indicates the grain size. The Hall–Petch relationship indicates that the grain size of metallic materials is inversely related to yield stress; thus, a smaller grain size corresponds to a higher yield strength of the material. Consequently, researchers have focused on grain refinement to enhance strength by reducing grain size. Subsequent studies have shown that grain refinement not only significantly improves yield strength but also maintains good ductility. Yang et al. [29] demonstrated that the refinement of magnesium alloy grains elevates the ratio of grain boundaries, impeding dislocation movement and consequently augmenting the material’s strength. Concurrently, the smaller grains offer additional pathways for dislocation slip, resulting in more uniform deformation. Liu et al. [24] show that fine-grained ZK60 alloys are more prone to activate basal plane slip and diminish reliance on twin deformation, and therefore attain a more balanced and robust plastic response [65]. Chen et al. [27] indicated that, in AZ31 alloys, twin-induced recrystallization and grain boundary bowing collaboratively contribute to significant microstructural refinement.

Grain refining can be classified into three primary categories. The first category is severe plastic deformation (SPD), including equal channel angular pressing (ECAP), high-pressure torsion, and repeated upset extrusion. Under substantial plastic deformations, high-density dislocations accumulate, triggering DRX and forming ultrafine or nanocrystalline structures [36,61,62,63,66]. For example, ECAP treatment of an RZ5 magnesium alloy can reduce grain size from several hundred microns to approximately ten microns while preserving ductility. The second category is heat-processing-induced DRX. By synergistically adjusting the deformation temperature, strain rate, and strain, the nucleation and growth processes of DRX can be regulated to obtain fine and uniform grains. Chen et al. [27] indicated that in the AZ31 alloy, twinning-induced recrystallization and the bowing of grain boundaries interact synergistically, leading to substantial refinement of the microstructure. The third category is alloying and trace element addition. Rare earth elements (e.g., Gd, Y, Zr) inhibit grain boundary migration and promote new grain nucleation, jointly contributing to grain refinement. When the grain size of magnesium alloys is reduced to below 0.1 μm (nanocrystals), twinning formation is inhibited due to the small grain size. Moreover, grain boundary slip becomes the predominant mode of deformation, leading to brittle fracture during elastic deformation. The current fabrication of magnesium alloys, with excellent mechanical characteristics via grain refinement, remains highly challenging, particularly as it relates to balancing the strength and ductility of nanocrystalline alloys.

### 4.2. Second-Phase and Twin Strengthening

Accurate design and precipitation control of the second phase have become essential for enhancing magnesium alloys. Second-phase strengthening mainly relies on the obstructive effect of fine, uniformly distributed second-phase particles on dislocation slip, while dispersed particles and grain boundary pinning mechanisms improve structural thermal stability [18,39,62,63,67,68,69]. Precipitation phases (e.g., Mg_17_Al_12_, Mg-RE phases) typically form during aging, inhibiting dislocation motion via the Orowan mechanism [70]. The strengthening effect is particularly significant when particles are of moderate size and uniformly dispersed [71]. Daghigh et al. [62] achieved synergistic fine-grain and precipitation strengthening of a WE43 alloy via ECAP and aging, achieving a strength of 410 MPa. Wang et al. [39] pointed out that the Mg_3_Bi_2_ particles in the AZ31 alloy can effectively pin grain boundaries and delay softening. Zhou et al. [72] found that the load transfer effect of the β-phase in the α-Mg matrix can enhance ductility.

The twin boundary not only reduces the mean free path of dislocations, enhancing the material’s strength, but it also inhibits dislocation movement, increasing work-hardening capacity. Twins can enhance plasticity during formation and provide slip channels for dislocations after formation, thereby improving the material’s ductility. In recent years, researchers have focused on nano-twinning to enhance the mechanical properties of a material. Lu [73] fabricated nano-twinned 304 stainless steel, achieving simultaneous strengthening and ductility improvement. As magnesium alloys have a limited number of easily activated slip systems, deformation twinning is a key plastic deformation mechanism [74,75]. Nano-twinning has been shown to enhance the strength and ductility of magnesium alloys [76]. Zhao et al. [77] fabricated magnesium alloys with twins through pre-deformation and subsequent heat treatment, achieving better mechanical properties than conventional alloys. Microstructural analysis showed that the interaction between dislocations and twin boundaries enhanced the materials’ strength and strain hardening capability. However, a key remaining challenge is controlling twin density and distribution, since excessive twinning reduces ductility and insufficient twinning fails to provide effective strengthening. Most studies focus on static twinning, with limited research on dynamic twinning under high-strain-rate compression, which is critical for automotive and ballistic applications.

### 4.3. Gradient and Heterogeneous Structures

Numerous natural materials (e.g., shells, turtle shells, bones) have multilayer composite structures: a rigid surface resists external impact and a softer inner layer absorbs impact energy. Mechanical properties are optimized via this unique structure [78,79]. Inspired by this, researchers have adopted gradient and heterogeneous structure designs to overcome the performance limitations of conventional homogeneous material systems [80]. The gradient structure depends on the incremental spatial variation in grain size, dislocation density, or texture. Heterogeneous structures include various typical variations, such as laminate structures, bimodal structures, heterogeneous lamellar structures, and composite-like heterogeneous architectures. The gradient structure induces strain gradients and back stresses through the cooperative deformation of variants, thereby enhancing the stress. Conversely, the heterostructure facilitates stress coordination through the differential deformation between soft and hard regions, significantly improving ductility [36,81,82,83,84,85,86].

Gradient microstructures in magnesium alloys can be fabricated using surface mechanical abrasion, radial forging, or additive manufacturing. In particular, additive manufacturing provides unique advantages in constructing gradient and heterogeneous structures due to its high design freedom, controllable thermal history, and ability to realize layer-by-layer deposition with tunable microstructures. By adjusting processing parameters, additive manufacturing can flexibly generate gradient grain structures, heterogeneous phase distributions, and gradient twin structures, making it a promising method for developing high-performance magnesium alloys with tailored mechanical properties.

Under external loading, the nanocrystalline surface layer of the gradient structure provides high strength, but its limited dislocation storage capacity due to the small grain size tends to cause localized plastic deformation. In contrast, the relatively coarse-grained interior suppresses strain localization in the surface nanolayer, helps maintain the structural integrity of the surface nanostructure, and preserves overall ductility. Meanwhile, the mismatched deformation between layers creates a strain gradient that contributes to additional strengthening and work hardening. As a result, gradient-structured magnesium alloys exhibit excellent comprehensive mechanical properties [81,82,83,84]. Heterostructured materials exhibit a similar strengthening mechanism. Under loading, hard domains bear the external load, while soft domains accommodate and dissipate strain. The strong dislocation interactions and strain redistribution between heterogeneous regions lead to heterogeneous deformation-induced (HDI) strengthening, which significantly improves yield strength and fracture toughness [85,86,87,88]. This effect has been verified in ZK60 and AZ91D alloys, which show enhanced work-hardening ability and suppressed crack propagation. Jiang et al. [87] investigated the effect of pre-deformation on the texture-dependent mechanical behavior of heterostructured magnesium alloy laminates. They found that pre-compression induced extension twinning and grain refinement, thereby improving mechanical properties along the extrusion direction.

Using microstructural heterogeneity to optimize macroscopic mechanical performance has become an effective strategy for developing high-performance magnesium alloys. This design concept provides a new route for achieving high strength and high toughness in engineering applications. However, most existing studies on gradient and heterostructured materials focus on face-centered cubic or body-centered cubic metals, while research on hexagonal close-packed magnesium alloys, especially their compressive mechanical properties, remains limited. Recent progress has begun to address this gap. Wang et al. [89] reported that gradient twin structures introduced in pure magnesium can interact with dislocations and cross twins during plastic deformation, thereby improving both strength and ductility. Zhang et al. [81] constructed a gradient structure with gradually varying grain size from fine to coarse in magnesium alloys. They revealed that gradient interfaces promoted the coordinated evolution of dislocations and twins under compression, leading to stepwise strain transfer and effective strain blocking. Consequently, both yield strength and uniform deformability were significantly enhanced. These results confirm that gradient layers can generate stable stress gradients in HCP magnesium alloys, thereby balancing strength and ductility, and provide a promising strategy for developing high-performance gradient magnesium alloys.

## 5. Conclusions and Outlook

This paper systematically reviews the research progress on the compressive mechanical behavior of magnesium alloys, with emphasis on micro-deformation mechanisms, macroscopic compressive properties, and strengthening strategies relevant to engineering applications.

Key findings reveal that plastic deformation under compression is governed by the synergy and competition between dislocation slip and twinning. Tensile twinning accommodates c-axis strain, while basal slip is readily activated yet restricted by crystallographic orientation. Non-basal slip, activated by elevated temperature or rare-earth alloying, serves as the critical contributor to enhanced plasticity, which is essential for forming and load-bearing engineering scenarios. The external conditions of temperature and strain rate effectively regulate the activity of slip/twinning and dynamic recrystallization. Specifically, high temperature promotes non-basal slip and DRX improves ductility; low temperature favors deformation twinning leading to high strength; and high strain rate enhances strain hardening and refines microstructures, offering controllable routes for industrial processing. For structural design, grain refinement, second-phase strengthening, twin hardening, as well as gradient and heterogeneous structures collectively achieve the synergy of strength and ductility. Fine grains promote slip activation, precipitates and dispersed particles impede dislocation motion, and gradient/heterogeneous structures induce favorable strain partitioning and hetero-deformation-induced (HDI) hardening, providing practical paradigms for performance optimization in lightweight components.

Future research directions with clear engineering relevance are proposed as follows:

(1) Cross-scale coupling mechanisms: Establish quantitative correlations between atomic-scale slip/twinning events and macroscopic compressive responses via multiscale modeling to support accurate performance prediction.
(2) Deformation under complex service loads: Clarify the evolution of twinning and dislocation–twin interactions under cyclic, impact, and combined stresses to improve fatigue resistance and service safety of structural parts.
(3) Innovative structural design: Develop scalable preparation methods for texture gradients, dislocation density gradients, and multiphase heterogeneous systems to expand the achievable performance envelope for industrial components.
(4) Data-driven intelligent design: Integrate machine learning and multiscale simulation to build high-precision models for property prediction and process optimization, realizing an efficient data-to-design closed loop.
(5) Service behavior in extreme environments: Reveal the coupled mechanical–environmental mechanisms under biodegradation and high-temperature corrosion conditions to support applications in biomedical implants and green manufacturing.

Overall, the understanding of magnesium alloy deformation has evolved from a single-mechanism analysis to a multi-mechanism, competitive–cooperative framework. Additionally, performance design has advanced from traditional alloying and homogeneous structure optimization to gradient and heterogeneous structure regulation. By integrating experimental characterization, numerical simulation, and artificial intelligence, magnesium alloy development will achieve a leap from law exploration to precise design, strongly promoting the engineering application of lightweight high-performance metallic materials in transportation, aerospace, and biomedical engineering.

## Data Availability

No new data were created or analyzed in this study. Data sharing is not applicable to this article.
